# The Impact of Diet and Lifestyle on Gut Microbiota and Human Health

**DOI:** 10.3390/nu7010017

**Published:** 2014-12-24

**Authors:** Michael A. Conlon, Anthony R. Bird

**Affiliations:** CSIRO Food and Nutrition Flagship, Kintore Ave, Adelaide, SA 5000, Australia; E-Mail: tony.bird@csiro.au

**Keywords:** diet, lifestyle, gut, microbiota, health

## Abstract

There is growing recognition of the role of diet and other environmental factors in modulating the composition and metabolic activity of the human gut microbiota, which in turn can impact health. This narrative review explores the relevant contemporary scientific literature to provide a general perspective of this broad area. Molecular technologies have greatly advanced our understanding of the complexity and diversity of the gut microbial communities within and between individuals. Diet, particularly macronutrients, has a major role in shaping the composition and activity of these complex populations. Despite the body of knowledge that exists on the effects of carbohydrates there are still many unanswered questions. The impacts of dietary fats and protein on the gut microbiota are less well defined. Both short- and long-term dietary change can influence the microbial profiles, and infant nutrition may have life-long consequences through microbial modulation of the immune system. The impact of environmental factors, including aspects of lifestyle, on the microbiota is particularly poorly understood but some of these factors are described. We also discuss the use and potential benefits of prebiotics and probiotics to modify microbial populations. A description of some areas that should be addressed in future research is also presented.

## 1. Introduction

There are approximately 10 times as many microorganisms within the gastro-intestinal (GI) tract of humans (approximately 100 trillion) as there are somatic cells within the body. While most of the microbes are bacteria, the gut can also harbor yeasts, single-cell eukaryotes, viruses and small parasitic worms. The number, type and function of microbes vary along the length of the GI tract but the majority is found within the large bowel where they contribute to the fermentation of undigested food components, especially carbohydrates/fiber, and to fecal bulk. Some of the most commonly found or recognized genera of gut bacteria in adults are *Bifidobacterium*, *Lactobacillus*, *Bacteroides*, *Clostridium*, *Escherichia*, *Streptococcus* and *Ruminococcus*. Approximately 60% of the bacteria belong to the *Bacteroidetes* or *Firmicutes* phyla [1]. Microbes which produce methane have been detected in about 50% of individuals and are classified as *Archaea* and not bacteria [2]. Although individuals may have up to several hundred species of microbes within their gut, recent findings from The Human Microbiome Project and others [3,4] show that thousands of different microbes may inhabit the gut of human populations collectively and confirm a high degree of variation in the composition of these populations between individuals. Despite this variation in taxa the abundance of many of the microbial genes for basic or house-keeping metabolic activities are quite similar between individuals [3]. There is growing evidence that imbalances in gut microbial populations can be associated with disease, including inflammatory bowel disease (IBD) [5], and could be contributing factors. Consequently, there is increased awareness of the role of the microbiota in maintaining health and significant research and commercial investment in this area. Gut microbes produce a large number of bioactive compounds that can influence health; some like vitamins are beneficial, but some products are toxic. Host immune defenses along the intestine, including a mucus barrier, help prevent potentially harmful bacteria from causing damage to tissues. The maintenance of a diverse and thriving population of beneficial gut bacteria helps to keep harmful bacteria at bay by competing for nutrients and sites of colonization. Dietary means, particularly the use of a range of fibers, may be the best way of maintaining a healthy gut microbiota population. Strategies such as ingestion of live beneficial bacteria (probiotics) may also assist in maintaining health. In this review, we will expand upon these subjects relating to diet and lifestyle, the gut microbiota and health, and provide some indication of opportunities and knowledge gaps in this area.

## 2. Microbial Products that Impact Health—Beneficial and Harmful

Microbial mass is a significant contributor to fecal bulk, which in turn is an important determinant of bowel health. Consumption of dietary fibers reduces the risk of colorectal cancer (CRC) [6] at least partly as a consequence of dilution and elimination of toxins through fecal bulk, driven by increases in fermentative bacteria and the presence of water-holding fibers [7,8,9]. Aspects of this will be discussed in more detail later in the review.

Gut microbes are capable of producing a vast range of products, the generation of which can be dependent on many factors, including nutrient availability and the luminal environment, particularly pH [10]. A more in-depth review of gut microbial products can be found elsewhere [11]. Microbial products can be taken up by GI tissues, potentially reach circulation and other tissues, and be excreted in urine or breath. Fermentation of fiber and protein by large bowel bacteria results in some of the most abundant and physiologically important products, namely short chain fatty acids (SCFA) which act as key sources of energy for colorectal tissues and bacteria, and promote cellular mechanisms that maintain tissue integrity [12,13,14]. SCFA can reach the circulation and impact immune function and inflammation in tissues such as the lung [15]. However, some protein fermentation products such as ammonia, phenols and hydrogen sulphide can also be toxic. There are many other products which deserve mention for their influence on health. Bacteria such as *Bifidobacterium* can generate vitamins (e.g., K, B_12_, Biotin, Folate, Thiamine) [11]. Synthesis of secondary bile acids, important components of lipid transport and turnover in humans, is mediated via bacteria, including *Lactobacillus*, *Bifidobacterium* and *Bacteroides* [11]. Numerous lipids with biological activity are produced by bacteria, including lipopolysaccharide (LPS), a component of the cell wall of gram negative bacteria that can cause tissue inflammation [16]. Also, many enteropathogenic bacteria (e.g., some *E. coli* strains) can produce toxins or cause diahorrea under the right conditions, but under normal circumstances other non-pathogenic commensal bacteria with similar metabolic activities outcompete and eventually eliminate them [17]. Bacteria such as *Bifidobacterium* can also help prevent pathogenic infection through production of acetate [18].

Many enzymes produced by microbes influence digestion and health. Indeed, much of the microbial diversity in the human gut may be attributable to the spectrum of microbial enzymatic capacity needed to degrade nutrients, particularly the many forms of complex polysaccharides that are consumed by humans [19]. Some bacteria such as *Bacteroides thetaiotamicron* have the capacity to produce an array of enzymes needed for carbohydrate breakdown [20], but in general numerous microbes appear to be required in a step-wise breakdown and use of complex substrates. Bacterial phytases of the large intestine degrade phytic acid present in grains, releasing minerals such as calcium, magnesium and phosphate that are complexed with it [21], making these available to host tissues (e.g., bone). Enzymes which degrade mucins help bacteria meet their energy needs and assist in the normal turnover of the mucus barrier lining the gut.

Competition between bacteria for substrates has a significant influence on which products are generated. Hydrogen is used by many bacteria and there is a hydrogen economy within the gut based around production by some bacteria and its use by others, including methanogens and sulphate-reducing bacteria (SRB) [22,23]. The use of hydrogen for production of methane by methanogenic *Archaea* may limit acetate production by other microbes, thereby potentially limiting production of beneficial butyrate and impacting health [2,23]. The role of methanogens in health is not yet clear. Breath methane correlates with levels of constipation in irritable bowel syndrome (IBS) [24] but methanogens numbers are depleted in IBD [2].

Production of gases such as methane, hydrogen, hydrogen sulphide and carbon dioxide is associated with digestion and fermentation within the GI tract. While excess production may cause GI problems such as bloating and pain, the gases may serve useful purposes. However, there is debate over whether hydrogen sulphide is largely beneficial or detrimental [23].

There is a strong interaction between the host immune system and the microbiota, with both producing compounds that influence the other. Some bacteria such as the key butyrate-producer *Faecalibacterium prausnitzii* may produce anti-inflammatory compounds [25]. Microbes also produce substances that allow communication between each other.

## 3. Lifestage and Lifetstyle Impacts on the Microbiota and the Influence of Nutrition

### 3.1. Lifestage

Microbes colonise the human gut during or shortly after birth. The fact that babies delivered naturally have higher gut bacterial counts at 1 month of age than those delivered by caesarean section [26] suggests gut colonization by microbes begins during, and is enhanced by, natural birth. The growth and development of a robust gut microbiota is important for the development of the immune system [27] and continues during breast-feeding, a stage which seems to be important for the long-term health of the individual. Oligosaccharides present in breast milk promote the growth of *Lactobacillus* and *Bifidobacterium*, which dominate the infant gut, and this can strengthen or promote development of the immune system and may help prevent conditions such as eczema and asthma [28,29,30]. These bacteria are undetectable in the stool of preterm infants in their first weeks of life [31]. A significant shift in the populations of gut microbes occurs when infants switch to a more solid and varied diet, including a decline in populations of *Lactobacillus* and *Bifidobacterium* to only a small percentage of the large bowel microbiota [32]. A wide diversity of microorganisms is needed to utilize the many fibers and other nutrients present in adult diets [19,33]. Functional maturation of the human microbiota, including the capacity to produce vitamins, increases during the early years of life [34].

The complexities and variability of adult gut microbial populations have become increasingly evident in recent years. The variability may relate to the influence of numerous factors, including diet and host genetics. The composition and activity of gut bacteria can vary according to (and possibly a result of) life events, including puberty, ovarian cycle, pregnancy and menopause [11]. The diets of children being weaned may have particular influence on microbial diversity in later life. Another broad shift in gut microbe populations occurs with age. The *Bacteroidetes* phylum bacteria tend to dominate numerically during youth but numbers decline significantly by old age, whereas the reverse trend occurs for bacteria of the *Firmicutes* phylum [11]. The consequences and reason for this change are not yet clear. However, the gut microbiota profiles of the elderly may not be optimal. One study found a high prevalence of potentially toxic *Clostridium perfringens* and lower numbers of *Bifidobacterium* and *Lactobacillus* in those in long-term care [35]. The latter also have a reduced microbial diversity compared to the elderly living in the community and this is related to increased frailty and changes in nutrition [36].

### 3.2. Lifestyle

The impact of non-dietary lifestyle factors on the gut microbiota has been largely ignored. Smoking and lack of exercise can significantly impact the large bowel (and potentially the microbiota) as they are risk factors for CRC [37]. Indeed, smoking has a significant influence on gut microbiota composition, increasing *Bacteroides-Prevotella* in individuals with Crohn’s Disease (CD) and healthy individuals [38]. Smoking-induced changes in microbial populations could potentially contribute to increased risk of CD. Air-borne toxic particles can reach the large bowel via mucociliary clearance from the lungs, and increased environmental pollution associated with industrialization could contribute to concomitant increases in IBD cases [39].

Another lifestyle factor, stress, has an impact on colonic motor activity via the gut-brain axis which can alter gut microbiota profiles, including lower numbers of potentially beneficial *Lactobacillus* [40]. Stress may contribute to IBS, one of the most common functional bowel disorders, and the associated changes in microbial populations via the central nervous system (CNS). The gut-brain axis is bi-directional, involving both hormonal and neuronal pathways [41], and so changes in the gut microbiota may influence brain activity, including mood [42]. Autism, a neurodevelopmental disorder, is associated with significant shifts in gut microbiota populations [43,44,45].

Obesity is associated with excess energy intakes and sedentary lifestyles. Exercise (or rather a lack of it) may be an important influence on any shifts in microbial populations that are associated with obesity. This is highlighted by a recent study that showed an increase in the diversity of gut microbial populations in professional athletes in response to exercise and the associated diet [46]. In humans and animal models with obesity, shifts in gut microbial populations occur, with increases in the *Firmicutes* and decreases in the *Bacteroidetes*, which could potentially contribute to adiposity through greater energy harvest [47,48,49]. However, other data suggests the shifts in microbial populations are driven primarily by the high fat obesogenic diets [50,51]. Irrespective of the cause, there are associated increases in gut bacteria linked with poor health outcomes (e.g., *Staphylococcus*, *E. coli*, *Enterobacteriaceae*) [52,53]. Dietary saturated fats may increase numbers of pro-inflammatory gut microbes by stimulating the formation of taurine-conjugated bile acids that promotes growth of these pathogens [54].

Geography also has a strong bearing on the composition of gut microbial populations. The diversity of fecal microbes in children from rural Africa is greater than that of children of developed communities in the EU, as is the number of bacteria associated with breakdown of fiber [55], suggesting dietary differences contributes significantly to the microbial differences. In another study, the type of fecal bacteria and their functional genes differed between individuals in the USA and in rural areas of Venezuela and Malawi [34].

Other environmental factors may also influence health via gut microbes. Travel, particularly to overseas destinations, increases the risk of contracting and spreading infectious diseases, including those causing diarrhoea. Some infections may go undiagnosed but result in long-term GI problems, including IBS [56]. Poor sanitary conditions in developing countries, and poor personal hygiene, can facilitate the spread of infectious agents. Circadian disorganization, occurring because of travel, shift work or other reasons, also impacts gut health and alters gut microbial populations [57].

## 4. Impacts of Macronutrients on the Gut Microbiota and Relevance to Health

### 4.1. Substrate Supply to the Colonic Microbiota

An adult colon contains approximately 500 g of contents, most of which is bacteria [58], and about 100 g/day is voided as stool. A typical western type diet supplies the colonic microbiota with about 50 g daily of potentially fermentable substrate, predominantly dietary fiber (DF). Non-starch polysaccharides (NSP) are major components of DF and account for 20%–45% of the dry matter supplied to the colon. Simple sugars and oligosaccharides each represent a further 10% whereas starch (and starch hydrolysis products) supplies less than 8% of dry matter. Some sugar alcohols also escape small intestine (SI) absorption and are minor dietary substrates for the colonic microbiota [59]. About 5–15 g of protein and 5–10 g of lipid passes into the proximal colon daily, largely of dietary origin. Various other minor dietary constituents, including polyphenols, catechins, lignin, tannins and micronutrients also nourish colonic microbes. About 90% of the approximately 1 g/day of dietary polyphenols escapes digestion and absorption in the SI [60,61] and can have significant influence on microbial populations and activities [62,63,64].

### 4.2. Carbohydrates—Importance for Large Bowel Fermentation and Health

Carbohydrates are the principal carbon and energy source for colonic microbes. Collectively, they have an immense capacity to hydrolyse a vast range of these nutrients, especially complex polysaccharides [65].

DF is integral to a healthy diet and Australian adults consume ~27 g each day [66], which is greater than in other high income countries, including the USA (<20 g/day). Epidemiological and experimental studies show that DF is both preventative and therapeutic for many large bowel disorders and other conditions or diseases, including cardiovascular diseases, type II diabetes and obesity [67,68,69,70,71].

One mechanism by which fiber promotes and maintains bowel health is through increasing digesta mass. Incompletely fermented fiber (e.g., insoluble NSP such as cellulose), increases digesta mass primarily though its physical presence and ability to adsorb water. An increase in digesta mass dilutes toxins, reduces intracolonic pressure, shortens transit time and increases defecation frequency. Fibers can also increase fecal mass to a lesser degree by stimulating fermentation, which leads to bacterial proliferation and increased biomass [7].

Many of the health benefits ascribed to fiber are a consequence of their fermentation by the colonic microbiota and the metabolites that are produced. Carbohydrates are fermented to organic acids that provide energy for other bacteria, the bowel epithelium and peripheral tissues. SCFA are the major endproducts of carbohydrate fermentation. These weak acids (pKa ~4.8) help lower the pH within the colon thereby inhibiting the growth and activity of pathogenic bacteria. Other minor organic acids produced include lactate, succinate and formate. Branched-chain SCFA (e.g., isobutyrate and isovalerate) results from fermentation of branched chain amino acids [72].

There are spatial gradients in microorganisms along the length of the gut. Bacterial growth and metabolic activity (fermentation) is greatest in the proximal colon where substrate availability is at a maximum [13,73]. Accordingly, pH progressively increases as stool progresses from the proximal to distal colon (from 5.8 to 7.0–7.5), largely because of the progressive depletion of carbohydrate substrates and absorption of SCFA, and increasing efficiency of protein fermentation and production of alkaline metabolites [72]. Total SCFA concentrations are highest in the proximal colon (~100 mM) and decline progressively toward the distal colon. Acetate, propionate and butyrate are the major individual SCFA, accounting for 90% of the total, with molar ratios approximating 65:20:15 [74].

Butyrate has attracted significant attention because it serves as the principal source of metabolic energy for the colonocytes [75], is instrumental in maintaining mucosal integrity, modulates intestinal inflammation and promotes genomic stability. The capacity of butyrate to regulate colonocyte differentiation and apoptosis, promoting removal of dysfunctional cells, underscores its potential to protect against colon cancer [76].

The SCFA also have roles beyond the gut and may improve risk of metabolic and immune system diseases and disorders, such as osteoarthritis, obesity, type II diabetes and cardiovascular disease [13,76].

More than 90% of the total SCFA produced in the colon is absorbed by the epithelium, through mechanisms that are not fully elucidated. SCFA-stimulated sodium-coupled transport in the apical membrane of colonocytes is especially important as it mediates (co)absorption of water and helps recover electrolytes as well as energy [77]. The SCFA can bind to G-protein coupled receptors in colorectal tissues, particularly GPR 41 and 43, which may influence immune function and tumour suppression, but these pathways are still relatively poorly characterized [76].

Most of the absorbed acetate reaches the liver via the portal vein, whereas propionate, and butyrate to an even larger extent, is metabolized extensively by colonocytes. Acetate and propionate are used by the liver for oxidation, and for lipogenesis and gluconeogenesis, respectively. Hepatic metabolic clearance of SCFA is very high and so concentrations in the systemic bloodstream are about 100-fold lower than those in colonic digesta and feces (~50 µM *versus* 100 mM, respectively) [13].

### 4.3. Protein

Dietary proteins are an important part of a balanced diet. Humans are unable to synthesize numerous amino acids and must obtain them from proteins in food to maintain health. Some protein-rich foods such as meat, eggs and nuts are also good sources of vitamins or nutrients such as iron. There is good evidence that a diet containing moderate to high amounts of protein can also contribute to weight loss in overweight individuals, particularly if combined with exercise [78], thereby minimising the health risks associated with obesity. Dietary proteins also have a significant impact on gut health. Depending on the type of protein and the other nutrients present in the food this can be beneficial or harmful. Some epidemiological studies, particularly large studies (up to 500,000 people), indicate a slight but significant association between CRC risk and the consumption of high levels of red and processed meats [79,80,81,82]. Not all epidemiological studies show such an association and the inconsistent findings may relate to the many factors which may contribute to CRC [83,84].

The potential for protein to harm colorectal tissues is explicable using current knowledge. An increase in protein intake usually results in more of the macronutrient, and hence fermentable substrate, reaching the colon. Although protein digestibility has an important influence on how much reaches the colon, most common dietary protein sources are highly susceptible to hydrolysis by SI enzymes. Dietary protein serves as the major source of nitrogen for colonic microbial growth and is essential to their assimilation of carbohydrates and the production of beneficial products such as SCFA. Hence, a combination of protein and carbohydrates in the large bowel can contribute to bowel health. However, unlike carbohydrates, fermentation of protein sources by the microbiota produces a much greater diversity of gases and metabolites, and increasing the nitrogenous substrate for the microbiota can also increase putrefactive fermentation products [85]. As digesta passes down the bowel its carbohydrate content dwindles and protein fermentation becomes progressively more important. Putrefactive fermentation has been implicated in the development and progression of many common bowel diseases given their greater prevalence in the distal colon [86], including CRC and IBD. Many of these protein fermentation endproducts, which include ammonia, hydrogen sulphide, amines, phenols, thiols and indoles, have been shown to be cytotoxins, genotoxins and carcinogens [87], in *in vitro* and animal models [88]. Generally, fecal levels of protein fermentation products, such as sulphide, are positively associated with dietary protein consumption in humans and there is evidence from rat studies that higher dietary protein intake (including higher red meat intake) is associated with greater DNA damage in colonic mucosa when dietary levels of fermentable carbohydrate are low [88,89,90,91]. Recently completed studies suggest that this relationship holds true for humans [92,93,94]. However, higher protein intake does not always result in higher fecal levels of protein fermentation products [95] nor does it necessarily increase the genotoxicity of fecal water in humans [96].

Although ammonia is a well-known toxin [97] it is used as an *N* source by the microbiota and most is excreted via stool or absorbed in the gut and eliminated in urine. Diets promoting microbial protein synthesis (and concomitant increased utilisation of ammonia), effectively reroute systemic *N* excretion from the kidneys to the fecal stream, which has benefits for renal health [98]. Other components derived from dietary protein sources such as red meat may also influence the gut microbiota and health. Microbial metabolism of l-carnitine, abundant in red meat, may generate products such as trimethylamine-*N*-oxide that could increase risk of atherosclerosis [99].

### 4.4. Fat

Dietary fat also influences the composition and metabolic activity of the gut microbiota and some evidence for this has been described earlier in relation to obesity.

High fat diets induce increased circulating levels of bacteria-derived LPS in humans, possibly as a consequence of increased intestinal permeability [100]. LPS is an immune system modulator and potent inflammatory agent linked to the development of common metabolic diseases.

The influence of dietary fat on the gut microbiota may be indirectly mediated by bile acids. Hepatic production and release of bile acids from the gall bladder into the SI, and the amount that escapes enterohepatic recycling and enters the colon, is increased with fat intake. Secondary bile acids, produced by 7 α-dehydroxylation of primary bile acids by colonic microbiota, are potentially carcinogenic and have been implicated in the aetiology of CRC and other GI diseases [101,102]. Further research is required on the interactions between dietary fat, the type and amount of bile acids that reach the large bowel, and the population structure and function of the microbiota in that viscus.

## 5. Effects of Polyphenols on the Microbiota

Dietary polyphenols, sourced from many foods including grapes, grains, tea, cocoa and berries, generally promote health and are linked to prevention of diseases such as cancer and cardiovascular disease [103]. Although many dietary polyphenols may have biological impacts through anti-oxidant effects or anti-inflammatory pathways [103], polyphenols which reach the colon can be metabolized by the resident microbiota and result in bioactive products, but our understanding of the microbial bioconversion processes is limited [104,105,106]. Metabolic profiling of polyphenolic products in excreta and blood using tools such as NMR is enabling greater insights into effects of dietary polyphenols in humans [107] but linking the metabolic changes to health outcomes remains a challenge [108]. Individual differences in microbiota populations may result in different capacities for polyphenol bioconversion [109] with potential consequences for health. In this context, it is noteworthy that the gut microbiota population profiles of individuals with IBD are significantly different from healthy individuals, and also that the polyphenolic metabolite profiles are also different between the two groups [110].

## 6. Western-Style Diets

The Western lifestyle, including diet, is associated with high incidences of chronic diseases, such as cardiovascular disease, CRC and type II diabetes which individually and collectively carry a hefty socioeconomic burden [111]. Most Western populations over-consume highly refined, omnivorous diets of poor nutritional quality. Those diets are energy dense, high in animal protein, total and saturated fats, and simple sugars but low in fruits, vegetables and other plant-based foods. Consequently, they are typically low in DF, NSP in general and RS in particular. For Western civilisations, refined cereal products (e.g., white bread) are the main DF source. Overfeeding (and sedentary behaviour) is also a hallmark of these populations.

Much of what is known about the diversity and complexity of human gut microbiota comes from molecular analysis of fecal samples obtained mainly from small cohorts of Caucasian adults habitually consuming Western style diets. Considerably less is understood about how other dietary patterns (e.g., vegetarian, Mediterranean) might influence the community structure and metabolic activity of microbiota.

## 7. Diet and Dietary Change

In humans, the microbial gene set is 150 times larger than the gene complement of the host [112]. However, only about 50 species belonging to just five or six genera and two phyla account for 99% of biomass. Of the genera *Bacteroides*, *Bifidobacterium* and *Eubacterium* are numerically the most important and may account for more than 60% of culturable bacteria present in human stool. *Clostridium*, *Enterobacteriaceae* and *Streptococcus* are also important but less numerous. Nearly all (~90%) of the bacteria in the human gut can be mapped to just two phyla, *Bacterioidetes* and *Firmicutes*. The relative proportions of the two dominant phyla vary, and can be influenced by a range of factors, but most people have similar proportions of each [113].

Long-term, habitual diet (*i.e.*, dietary pattern) and shorter term dietary variation influences gut microbiota composition. The population structure is responsive to acute dietary change (daily variation), as evidenced by rapid and substantial increases in populations at the genus and species level. However, dietary change does not necessarily result in a permanent (paradigm) compositional shift, at least at phylum level, although evidence for this assertion is limited [114].

## 8. Dietary Patterns, Macronutrients and Microbiota Taxonomic Composition

### 8.1. Observational Studies

Cross-sectional studies have shown some evidence that Western-style diets are associated with gut microbial populations that are typified by a *Bacteroides* enterotype whereas traditional diets rich in plant polysaccharides are associated with a *Prevotella* enterotype [114]. The *Prevotella* enterotype was only weakly associated with components that typify Western diets but strongly linked to carbohydrates and simple sugars. The fecal microbiota of children in the USA is dominated by *Bacteroides* [34,115]. Similarly, Italian children have high levels of *Enterobacteriaceae* (mainly *Shigella*, *Escherichia* and *Salmonella*). In contrast, the stool of children in rural Africa and South America consuming traditional plant-based diets was enriched in *Bacteroidetes*, in particular the *Prevotella* enterotype and species associated with fiber utilization (e.g., *Xylanibacter*) [55]. *Prevotella* and (*Xylanibacter*) are known to use cellulose and xylans as substrates [55,116]. Diets of North American and Italian urban children are much richer in animal protein and saturated fats whereas the diets for the other two populations are plant-based and have higher levels of fiber. The *Bacteroidetes:Firmicutes* ratio was lower for children in the Western countries.

As stated earlier, there is a paucity of data on the association between vegetarian dietary patterns and the gut microbiota, especially using molecular methods. A study that used PCR-denaturing gradient gel electrophoresis (DGGE) for microbial population fingerprinting found no significant differences in the fecal microbiota of vegetarians and omnivores, although the abundance of *Clostridium* cluster IV in the latter tended to be greater [117]. In a cohort of female college students from rural India, the fecal microbiota of those whose dietary pattern was omnivorous had a greater relative abundance of *Clostridium* cluster XIVa bacteria, specifically *Roseburia-E. rectale* (butyrate-producing bacteria), compared to the lacto-vegetarians [118]. There were no differences in the relative proportions of other major bacterial groups targeted. A gene encoding for a pivotal enzyme (butyryl-CoA CoA-transferase) involved in butyrate synthesis was also upregulated in the omnivores. The study demonstrates differences in the composition and functional capacity of the microbiota of individuals with two markedly diverse dietary patterns.

The taxonomic diversity of the fecal microbiota of individuals on habitual Western diets appears to be less than for those consuming plant-based diets. Also, individuals who are obese or have type II diabetes, inflammatory diseases (osteoarthritis) and other major health problems (prevalent in Western societies) have a sub-optimal fecal microbiota profile. Specifically, it is less diverse than that of healthy controls [119,120] and there are also major compositional differences at the phylum level. Obesity is associated with an increased fecal *Bacteroidetes:Firmicutes* ratio relative to lean subjects [121]. Whether a microbiota with lower compositional diversity is less resilient to environmental challenges and is less “healthier” for the host is not yet known [122].

The fecal hydrogenotrophic microbiota of native Africans, whose diet is low in animal products, compared to that of African and European Americans consuming a typical Western diet was more diverse and contained different populations of hydrogenotrophic *Archaea* and methanogenic *Archaea* as well as SRB populations [123]. The differences in bacterial community structures of native African populations were reflective of the diets of the hosts. Those on Western diets, characterized by higher intakes of dietary animal proteins (as meat, milk and eggs), may deliver greater amounts of sulphur compounds to the colonic microbiota [124], thus favouring sulfidogenic hydrogen disposal whereas in native Africans methane is the major hydrogen sink. Native African populations have lower intake of animal products and higher breath methane concentrations than westernized populations [123,125].

### 8.2. Dietary Interventions

Replacing a habitual Western diet with one high in fiber elicited rapid (within 24 h) and marked alterations in fecal microbiota composition, although the changes were insufficient to produce a broad switch from *Bacteroides* to *Prevotella* enterotype [114].

In an inpatient study [126], altering dietary energy load in lean and obese adults induced rapid changes in the proportional abundance of *Bacteroidetes* and *Firmicutes*. The former decreased whereas the latter increased with increasing energy intake. Further studies are required to determine if the changes in microbiota composition were the result of the increase in dietary fat or another macronutrient. High fat diets are also associated with substantial compositional changes in the colonic microbiota at the phylum and genus levels, including reductions in both Gram positive (e.g., *Bifidobacterium* spp.) and Gram negative bacteria (e.g., *Bacteroides*) [123].

Animal models are also proving useful in understanding factors that impact the gut microbiota, particularly in regards to high fat diets and obesity. A study using a murine (RELMβ) knockout model showed that dietary fat-induced changes to gut microbiome composition were independent of obesity [127]. In conventional mice, increased dietary fat intake resulted in fewer numbers of *Bacteroidetes* and increases in *Firmicutes* and *Proteobacteria*. A high fat diet also reduced cecal *Bifidobacterium* numbers and increased circulating LPS concentrations [128,129] and has also been shown to reduce the abundance of *Clostridium* cluster XIVa, including *Roseburia* spp. [130]. Diet-induced changes in mucosal integrity have been shown to promote metabolic endotoxemia and trigger systemic low grade inflammatory responses in a range of tissues [100,128,129].

## 9. Microbes and Mucosal Health

A layer of mucus, produced by goblet cells, lines the epithelium of the GI tract and acts as a barrier to microbial invasion of tissues and can contribute to intestinal homeostasis [131,132]. The basic component of mucus is mucin. Some bacterial products (SCFA) stimulate the production of mucus in response to dietary components such as NSP [133]. Over-utilization of the mucus by bacteria or reduced production can lead to thinning of the barrier under certain dietary conditions [88]. In the colon, “mucin-depleted foci” may develop as one of the features associated with tumorigenesis in rodents and humans in response to carcinogens [134]. However, the role of mucin depletion in oncogenesis is not clear as a recent study in rats showed that inflammation associated with mucin-depleted foci was not due to infiltration of bacteria, whereas colonic tumors did appear to be colonized by bacteria [135]. Many bacteria can adhere to and degrade the outer layer of colonic mucus but the inner layer is generally bacteria free [136]. Although break-down of mucus by bacteria is a normal part of mucus barrier turnover, an overabundance of mucus-degrading bacteria, such as *Akkermansia muciniphila* in the adherent mucus layer of individuals with IBD [137,138], could contribute to tissue inflammation by weakening the barrier.

Tight junctions between cells also helps prevent translocation of bacteria and molecules (including toxins) across gut epithelial tissues. A loss of this integrity (a so-called “leaky gut”) may have serious consequence for health. In the first few years of life, interactions between the gut microbiota and the mucosal barrier appear important and perturbations in the relationship that lead to excessive gut permeability and immune changes may result in susceptibility to a range of diseases in later life [139]. A significant proportion of the activities of the immune system occur within the gut. Gut-associated lymphatics contribute substantially to this defense but other cells lining the gut also produce a range of molecules which can neutralise pathogenic microbes. Dendritic cells sample the gut luminal environment for harmful bacteria and can induce a suite of responses including the activation of macrophages, B cells and T cells within mucosal tissues and the release of broad specificity ant-microbial agents such as Immunoglobulin A and α-defensins into the luminal environment [140].

A loss of gut barrier function may contribute to numerous diseases. An example is Parkinsons disease (PD), a multi-system disease in which there is dysfunction of the GI tract, including changes in the enteric nervous system which appear before obvious degeneration of the CNS [141,142]. Individuals with PD have increased intestinal permeability, greater intestinal infiltration of *E. coli* and greater endotoxin (LPS) exposure, and these changes correlate with the enteric neuronal damage [143], leading to suggestions that a pathogen may be responsible for PD [144] and a breakdown in mucosal barrier function may play a central role. An impaired gut barrier may also contribute to symptoms or complications of autism, kidney disease, type 2 diabetes, cardiovascular disease, metabolic syndrome, obesity, and liver diseases [45,100,145,146,147,148,149].

## 10. Inter-Individual Variation in Gut Microbiota and Responses to Diet

Each individual has a distinct combination of gut microbial species. This has become increasingly evident from molecular analyses of recent decades, including The Human Microbiome Project. One metagenomic analysis also suggested that the gut microbiota of each human is typified by one of three enterotypes, with each enterotype characterised by distinct dominant groups of microbes [150], namely *Bacteroides*, *Prevotella* and *Ruminococcus*. However, subsequent studies, including those of The Human Microbiome Project, have been unable to provide clear support for the concept as initially proposed [4,114]. More recent findings and analysis of the evidence roughly support typing with *Prevotella* or *Bacteroides* dominance of the microbiota but the numerous factors, especially dietary, that impact gut microbial populations means there is considerable variation in numbers of these genera, making it difficult to classify populations as a particular “type” [113].

Inter-individual differences in populations of the gut microbiota may lead to different capacities to utilize dietary components and to different levels of disease risk. For example, some individuals have consistently low stool levels of the microbial fermentation product butyrate, levels which generally remain lower relative to others despite concentrations increasing in response to a diet high in RS [151]. Butyrate production is important for the maintenance of colorectal tissue integrity and may protect against colorectal diseases [13,76]. Individual differences in numbers and functions of bacteria such as *Ruminococcus bromii*, important for the generation of SCFA in response to RS in humans [152,153], could potentially influence colorectal health.

## 11. Use of Probiotics and Prebiotics as Nutritional Strategies to Improve Health

Probiosis and prebiosis are diet-based processes/strategies for promoting the health of the host through improving the composition of the colonic microbiota. Although both prebiotics and probiotics have been shown to increase numbers of selected bacteria at the species and genus level, typically *Bifidobacterium* and *Lactobacillus*, changes in the overall composition of the gut microbiota are often relatively small, and generally persist only for as long as the period of the intervention. Also, definitive proof that the identified compositional alterations are directly responsible for an improvement in host health generally remains elusive. While the concepts have practical relevance they are simplistic given the current limited understanding of the complex and dynamic interplay between the host and their gut microbiota.

Prebiotics are dietary substrates that selectively promote proliferation and/or activity of “beneficial” bacteria indigenous to the colon. The concept, first published by Gibson and Roberfroid [154] in 1995, has been refined and redefined on several occasions. Prebiotics are defined currently as “selectively fermented ingredients that result in specific changes, in the composition and/or activity in the GI microbiota, thus conferring benefit(s) upon host health” [155].

To qualify as a prebiotic all of the following properties must be demonstrated: (i) a food ingredient that escapes assimilation in the small intestine; (ii) upon reaching the colon its fermentation by the microbiota flora selectively alters its taxonomic composition and/or activity which (iii) confers demonstrable health benefits for the consumer [156].

The validity of the prebiotic concept and evidence of a role for prebiotics in promoting health and reducing risk of bowel and systemic diseases have been recently reviewed in depth [157,158,159,160]. Data from studies in animals provides strong evidence of the potential of prebiotics to afford protection against a range of chronic diseases or conditions common in humans (e.g., CRC, IBD, type 2 diabetes, obesity) by preventing colonization by enteric pathogens [61,158,161]. Prebiotics have been shown to improve bowel and immune function, metabolic health and mineral bioavailability in humans but the evidence is strong only for bowel habit and colonic uptake of calcium and magnesium. There is mounting evidence that prebiotics both directly and indirectly modulate the immune system and reduce the risk and severity of bowel infectious and inflammatory conditions, such as IBD, as well as functional bowel disorders, notably IBS [159].

Short-chain nondigestible carbohydrates (inulin-type fructans, fructo-oligosaccharides (FOS) and galacto-oligosaccharides (GOS)) are the quintessential prebiotics and the target bacterial groups are typically *Bifidobacterium* and *Lactobacillus*. Fructan prebiotics, such as inulin and FOS, occur naturally in various foods including cereals, fruits and vegetables and so are ubiquitous in most diets. Dietary intakes have been estimated to be ~5–10 g/day [162].

The prebiotic concept as it currently stands is probably too narrowly focused. It has been proposed [163,164] that the taxonomic focus should be widened beyond the *Bifidobacterium* and *Lactobacillus* which have been historical targets. These genera may not be the most important contributors to host health. Emerging candidates include *Ruminococcus bromii*, *Roseburia intestinalis*, *Eubacterium rectale*, and *Faecalibactrium prausnitzii*, but there are many others that may be of benefit. It has been suggested that a prebiotic index might offer greater utility for evaluating the efficacy of different prebiotics [165]. The prebiotic concept also encompasses selective improvements in metabolic activity of the microbiota but this has been given little attention to date. Changes in concentration patterns of key beneficial microbial metabolites such as butyrate should be integrated into prebiotic index models.

All established prebiotics to date are carbohydrates, specifically inulin type fructans and GOS. However, other dietary carbohydrates also qualify as prebiotics, for instance resistant starch (RS) [156,166], but evidence from human studies is limited. More studies are required on the prebiotic properties of different types, doses and food sources of RS. The inter-individual variability in the microbial response to RS suggests successful dietary interventions with RS need to be personalised [167]. Dietary constituents other than carbohydrates conceivably could function as prebiotics. For instance, cocoa flavonols can increase the relative abundance of *Bifidobacterium* and *Lactobacillus* at the expense of potentially pathogenic bacteria, notably the *C. histolyticum* group [64].

Probiotics are defined as live microorganisms which when administered in adequate amounts confer a health benefit on the host. The most commonly consumed probiotics belong to the genus *Lactobacillus* and *Bifidobacterium*. Mechanisms by which probiotics might improve host health include immune function augmentation through reinforcing mucosal barrier function, reducing mucosal transfer of luminal organisms and metabolites to the host, increasing mucosal antibody production, strengthening epithelia integrity and direct antagonism of pathogenic microorganisms. However, the results of studies in humans are varied, due most likely to methodological differences (dose and duration of probiotic administration, sampling regimen and microbiological techniques) and differences in host cohorts (age, health status). Perhaps most importantly, it is clear from *in vivo* studies in humans and animal models that probiotic efficacy in promoting health is strain dependent and not species and genus specific. For a more comprehensive and detailed description of the health benefits of probiotics and their prophylactic potential for various gut diseases the reader is referred to recent narrative and systematic reviews [168,169,170,171,172,173].

## 12. Gaps in Understanding

There are still many gaps in our understanding of the interactions between diet, lifestyle, gut microbes and health. Here, we present some of the areas we believe should be addressed to help fill that knowledge gap.

There is a growing need for an understanding of the activities of gut microbes, particularly their physiological relevance. Current molecular methods such as sequencing technologies are allowing the identification of the many hundreds of microbial species present in human GI tracts and are beginning to identify the types of genes that they possess. The next steps will be to understand the functions of the many poorly characterised microbes, particularly their roles in the breakdown of food and how the associated by-products contribute to health and disease.

The majority of gut microbes are present within the large bowel and most GI microbiology research has focused on this area. However, the SI can, like the colon and rectum, become inflamed in cases of IBD and bacteria are implicated. The role that bacteria play in SI enteropathies and leaky gut is also yet to be clearly elucidated. The contribution of diet to maintenance of SI health is also not well understood. Understanding in this area is hampered by the general inaccessibility to these sites within human subjects, especially in healthy individuals who have no need to visit a gastroenterologist.

The integrity of the gut mucosa is of critical importance to health. Understanding which foods or dietary components strengthen or weaken that barrier may assist in tailoring diets to prevent microbes and toxins such as LPS from accessing tissues and causing inflammation. A better understanding of the interactions between the host immune system and gut bacteria, particularly in children, should also shed light on how microbes may contribute to lifelong susceptibility to some diseases, and how diet may be used to promote optimal microbial populations.

The gut-brain axis is increasingly viewed as having an important role in health with bi-directional communication of information of relevance to areas such as satiety, mood and gut motility and suggestions of roles in conditions that include IBS and autism. The gut microbiota has been implicated in some of these conditions and there is great scope for research into understanding which of the many microbial products reach the CNS and impact health, including mental health, via the brain, and consequently for understanding how dietary manipulation of the microbiota then impacts these important areas.

Since it has been shown that many microbial products can influence health, the inter-individual variation in gut microbial profiles in humans may lead to differences in disease risk. A better understanding of the origins of the variation may ultimately allow the microbial profiles to be modulated. Environmental and dietary factors appear to play some role during a child’s early development but the extent to which host genetics contribute to the variation is not known. Studies which follow the development of microbial profiles in children, and the impact that diet and environment have on these, are sorely needed. It may be possible to develop diets that lead to optimal microbial population and health outcomes.

The extent to which long-term dietary patterns can shift the composition of microbial populations is yet to be clearly determined despite some emerging knowledge and should be investigated further. Our understanding of how different sources and forms of macronutrients such as carbohydrates, proteins and lipids interact and affect the GI tract is still lacking. Knowledge of how micronutrients impact the gut and its microbes is even scarcer.

Food structure is also an important determinant of how a food impacts the body, with particle size and the associated food matrix influencing the accessibility of host and microbial enzymes to nutrients. For example, smaller starch granules may be more readily degraded due to higher surface area to volume ratios and this increases the rate at which sugars are absorbed by tissues, an important issue when considering glycaemic control. Since most gut microbes are within the large bowel, food structures which minimize SI digestion and allow food to pass into the colon will have the greatest impact on the microbiota. Cooking practices can potentially impact food structure and digestibility of foods. Strategies which optimize the structure of foods for defined benefits (*i.e.*, glycemic control) are already being implemented but more work is needed in this area to achieve a broader range of health outcomes.

Ingestion of probiotics, prebiotics and microencapsulated nutrients, beneficial molecules or microbes [174,175] is designed to deliver a health benefit to the body by increasing numbers of beneficial microbes or their products with the gut. A greater knowledge of which microbes and functions are beneficial is needed to effectively culture, deliver and/or stimulate the growth of the appropriate microbes. Presently, only a small number of adult human gut microbes have been used for probiotics, or targeted in assessments of the impacts of diets (including prebiotics) on gut health.

## 13. Conclusions

We have sought to provide a broad picture of how diet, and to some extent lifestyle, can have significant and wide-ranging impacts on human health and shown that the microbes which inhabit the GI tract play an important role in mediating these effects. Although significant gains have been made recently in our understanding of the complexity of gut microbial populations, a more detailed understanding is needed of microbial functions and products that maintain (or negatively impact) the integrity of tissues, at sites within and distant from the gut. Alongside this is a need to understand which factors within the diet supply substrates to the microbes so that this knowledge can be harnessed to generate the desired shifts in microbial populations, products and health outcomes. Particularly challenging will be the task of understanding what constitutes a healthy population of gut microbes. Certain microbial population profiles may be associated with diseases and conditions. In many cases, it is not clear if environmental/lifestyle factors, diet or genetic predisposition leads to these profiles, or indeed whether the altered microbial populations contribute to the condition. While dietary intervention can induce significant change, it is possible that the level of impact may not always be sufficient to engineer the changes in microbial populations that are conducive to better health. The use of probiotics and other strategies may be required. An understanding of the ontogenesis of our gut microbial population profiles, and how this contributes to the development of our immune system, may enable early intervention or prevention of the formation of undesirable microbial profiles and the consequences. In this context, defining the factors which dictate the development of our human microbial populations in early life will be important.
